# A multistate model of survival prediction and event monitoring in prefibrotic myelofibrosis

**DOI:** 10.1038/s41408-020-00368-1

**Published:** 2020-10-14

**Authors:** Alessandra Carobbio, Paola Guglielmelli, Elisa Rumi, Chiara Cavalloni, Valerio De Stefano, Silvia Betti, Alessandro Rambaldi, Maria Chiara Finazzi, Juergen Thiele, Alessandro M. Vannucchi, Ayalew Tefferi, Tiziano Barbui

**Affiliations:** 1 FROM Research Foundation, Papa Giovanni XXIII Hospital, Bergamo, Italy; 2 Center Research and Innovation of Myeloproliferative Neoplasms (CRIMM), Department of Experimental and Clinical Medicine, Azienda Ospedaliera Universitaria Careggi, University of Florence, Florence, Italy; 3 Division of Hematology, Fondazione IRCCS Policlinico San Matteo, and Department of Molecular Medicine, University of Pavia, Pavia, Italy; 4 Institute of Hematology, Catholic University, Rome, Italy; 5 Fondazione Policlinico “A. Gemelli” IRCCS, Rome, Italy; 6 Hematology and Bone Marrow Transplant Unit, ASST Papa Giovanni XXIII, Bergamo, Italy; 7 Department of Oncology and Hematology, University of Milan, Milan, Italy; 8 Institute of Pathology, University of Cologne, Cologne, Germany; 9 Hematology Division, Mayo Clinic, Rochester, MN USA

**Keywords:** Prognosis, Myeloproliferative disease

## Abstract

Among 382 patients with WHO-defined prefibrotic myelofibrosis (pre-PMF) followed for a median of 6.9 years, fibrotic or leukemic transformation or death accounts for 15, 7, and 27% of cases, respectively. A multistate model was applied to analyze survival data taking into account intermediate states that are part of the clinical course of pre-PMF, including overt PMF and acute myeloid leukemia (AML). Within this multistate framework, multivariable models disclosed older age (>65 years) and leukocytosis (>15 × 10^9^/L) as predictors of death and leukemic transformation. The risk factors for fibrotic progression included anemia and grade 1 bone marrow fibrosis. The outcome was further affected by high molecular risk (HMR) but not driver mutations. Direct transition to overt PMF, AML, or death occurred in 15.2, 4.7, and 17.3% of patients, respectively. The risk of AML was the highest in the first 5 years (7%), but leveled off thereafter. Conversely, the probability of death from overt PMF or AML increased more rapidly over time, especially when compared to death in the pre-PMF state without disease progression. The probability of being alive with pre-PMF status decreased to 70 and 30% at 10 and 20 years, respectively. This study highlights the aspects of the clinical course and estimates of disease progression in pre-PMF.

## Introduction

Diagnostic criteria established by the World Health Organization (WHO)^1,2^ highlight the clinical relevance of distinguishing prefibrotic/early-stage primary myelofibrosis (pre-PMF) from both overtly fibrotic PMF and essential thrombocythemia (ET)^3–9^. The presenting clinical picture of pre-PMF is heterogeneous ranging from isolated thrombocytosis to systemic manifestation resembling overt PMF. Pre-PMF displays a progressive clinical course with development of anemia, enlarging spleen size, worsening of constitutional symptoms, increased grades of bone marrow (BM) fibrosis, and emergence of high-risk cytogenetic or molecular abnormalities; accordingly, pre- and overt PMF are assumed to represent a phenotypic continuum^10^.

This paper focuses on the rates of disease progression in pre-PMF taking into account the different phases of disease in its clinical course. To that effect, we considered 382 patients with pre-PMF diagnosed according to WHO criteria: we aimed (i) to evaluate transition estimates between the initial state of pre-PMF and the absorbing state, i.e., death, through the intermediate-state passages, (ii) to search prognostic factors predicting the transition of patients through different clinical conditions.

## Patients and methods

### Patients

This study collected retrospective data in 382 patients from four different tertiary Italian centers with large experience regarding myeloproliferative neoplasms, after the approval of all institutional ethical committees (first approval of the coordinating center Papa Giovanni XXIII’s Hospital, n.208-18) and informed consent of participants, according to the mandates of the Helsinki declaration.

Patients’ eligibility criteria included the revised 2016 WHO criteria^1^ until December 31, 2017, together with genotypes for *JAK2*V617F, *MPL*W515L/K, and *CALR* mutations. In a subgroup of patients (*n* = 132) referred to a single center participating in this study, high molecular-risk (HMR) mutations, i.e., *ASXL1, EZH2, SRSF2*, and *IDH1/2*, were also analyzed; a HMR category was defined by the presence of at least one mutation^11^. The median duration of follow-up was 6.89 years (range 0.08–32.6).

Diagnosis of progression to overt PMF was established by the corresponding clinical features. The latter included worsening of anemia, increase in splenomegaly, and/or overt leukoerythroblastosis consistent with advanced PMF with myeloid metaplasia. Transformation to AML met criteria (20% blasts) in agreement with the WHO classification^1,2^.

### Statistical methods

**Fig. 1:**
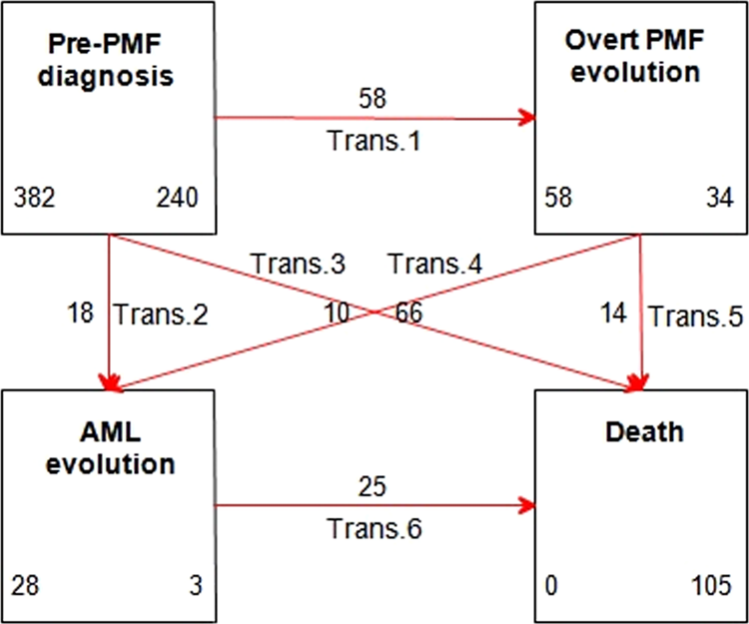
Outline of the multistate model of transition to overt PMF, AML, or death. Trans transition, AML acute myeloid leukemia, PMF primary myelofibrosis. The figure shows the 4-state model for the clinical course of pre-PMF patients. The four states, graphed by boxes, are (1) diagnosis of pre-PMF, (2) evolution into overt PMF, (3) evolution into AML, and (4) death. In each box is quoted the number of patients starting (left) and ending (right) in the state. Across states, the following six transitions (graphed by arrows) are possible: from pre- to overt PMF (trans.1), from pre-PMF to AML (trans. 2), from pre-PMF to death (trans. 3), from overt PMF to AML (trans. 4), from overt PMF to death (trans. 5), and from AML to death (trans. 6). On each arrow is quoted the number of patients involved in the corresponding transition.

A multistate model was applied to analyze data of survival, taking into account intermediate states that are part of the clinical course of pre-PMF, where patients may transit through evolution to overt PMF and/or AML (transient states) before death (absorbing state). The outline description of the adopted model is represented in Fig. 1. A parametric Markov multistate survival model was applied, according to the approach proposed by Crowther et al.^12^.

Within this multistate framework, in order to investigate risk factors for each specific transition between two states, different multivariable Cox regression models assuming a Weibull distribution of the baseline hazard, were performed for clinical and mutational covariates separately. Performances obtained by both models were then compared by values of the Akaike’s information criterion (AIC)^13,14^.

## Results

### Patient characteristics

A total of 382 patients with WHO-defined pre-PMF were included in this study; their clinical and hematologic characteristics at diagnosis are reported in Table 1. Median age was 57.6 years, 45% were above the age of 60; 51% were male. Generic cardiovascular risk factors for thrombosis were present in 45% of cases. Of note, grade 1 BM reticulin fibrosis was found in 58% of patients and palpable splenomegaly >5 cm in 15%. Forty percent of patients displayed LDH values that were >1.5 times the upper limit of normal, whereas 15% of informative patients displayed an abnormal karyotype.

**Table 1:** Clinical and mutational characteristics of 382 pre-PMF patients.

**Hematology and clinical features**	
*N* (%)	382
Male/female, *n* (%)	195/187 (51/49)
Age, years, median (range)	57.6 (15.6–91.9)
≥60 years, *n* (%)	170 (45)
*Blood values*	
Hb, g/dL, median (5th–95th percentiles)	13.5 (9.3–15.9)
Hct, g/dL, median (5th–95th percentiles)	41.1 (30.6–49.0)
Plt count, ×10^9^/L, median (5th–95th percentiles)	700 (130–1481)
WBC count, ×10^9^/L, median (5th–95th percentiles)	10.0 (5.0–24.1)
*Fibrosis grade*, *n* (%)	
0	161 (42)
1	221 (58)
Palpable splenomegaly, *n* (%)	176 (46)
Spleen size from LCM > 5 cm, *n* (%)	58 (15)
LDH > normal range, *n* (%)	220 (73)
LDH > 1.5 times the normal range, *n* (%)	119 (40)
CD34, median (range)	7.4 (0–6961)
Circulating blasts ≥1%, *n* (%)	19 (5)
Abnormal cytogenetics, *n* (%)	42 (15)
**Mutational landscape**	
*Driver mutations*	
*JAK2*V617F, *n* (%)	246 (65)
*Allele burden, median (range)*	*36.8 (0.3–100)*
*CALR* type1, *n* (%)	63 (22)
*Allele burden, median (range)*	*50 (9–63.7)*
*CALR* type2, *n* (%)	24 (18)
*Allele burden, median (range)*	*49 (7–94)*
*MPL*W515L/K, *n* (%)	17 (5)
Triple negatives, *n* (%)	31 (8)
*Nondiver mutations* (*N* = 132)	
*ASXL1*, *n* (%)	28 (21)
*EZH2*, *n* (%)	5 (4)
*SRSF2*, *n* (%)	14 (11)
*IDH1/2*, *n* (%)	1 (1)
HMR^a^, *n* (%)	37 (28)
HMR^b^ 2, *n* (%)	11 (8)

*Hb* hemoglobin, *Hct* hematocrit, *Plt* platelets, *WBC* white blood cell, *LDH* lactate dehydrogenase, *HMR* high molecular risk.

^a^HMR points to the presence of at least one mutation in any one of ASXL1, EZH2, SRSF2, and IDH1/2.

^b^HMR 2 means the presence of two or more mutated genes among the above. Two or more mutations in the same gene are counted as one.

The distribution of driver mutations was as follows: *JAK2*V617F in 65%, *MPL*W515L/K 4.5%, *CALR* type1 16.5%, *CALR* type2 6%, and triple negativity in 31 patients (8%). A total of 132 patients (35%) were screened for other additional mutations: HMR mutations were found in 28%, and *ASXL1* was the most frequent. Patients were classified as HMR for the presence of one (28%) or more than one (8%) mutation. Overall, 239 patients (77%) received cytoreductive treatment (hydroxyurea in 68%), antiplatelet agent in 72%, and oral anticoagulants and/or heparin in 15%.

### Multistate model for death and disease progression in pre-PMF

This model is composed of four states (pre-, overt PMF, AML, and death) and six possible transitions between states. Figure 1 illustrates each transition, as well as the number of patients starting and ending in each disease state. All patients begin in the initial pre-PMF state (*n* = 382), and then they can move directly to overt PMF (transition 1, *n* = 58), AML (transition 2, *n* = 18), or death (transition 3, *n* = 66). The remaining three transitions describe the movement of patients from an intermediate to the next (or final) state. In detail, patients who previously evolved into overt PMF, can progress to AML (transition 4, *n* = 10) or death (transition 5, *n* = 14); conversely, the only possible transition from AML is to death (transition 6, *n* = 25).

At the end of the study follow-up (median 6.89 years, range 0.08–32.6), 240 (63%) patients with pre-PMF remained alive and event free in the initial status; 34 (9%) and 3 (1%) patients of the initial pre-PMF cases, who progressed to overt PMF and AML, respectively, were alive, and 105 (27%) patients had died.

### Transition and state occupation probabilities

**Fig. 2:**
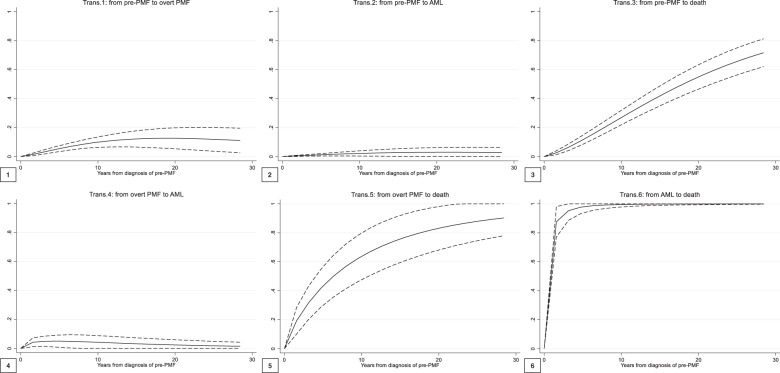
Transition probabilities over time from pre- to overt PMF, AML, and death (1, 2, and 3), from overt PMF to AML and death (4 and 5), and from AML to death (6). Trans transition, AML acute myeloid leukemia, PMF primary myelofibrosis. This figure shows the six transition probabilities of the multistate model for the clinical course of pre-PMF. Transition probabilities are defined as the probability of going from a given state to the next state in a Markov process. Direct transitions (panels 1–3) refer to all the 382 patients initially at risk (the pre-PMF state); thus, they represent the probability that a patient with a pre-PMF can directly evolve into overt PMF or AML or die. Panels 4–6 show the probabilities of indirect transitions, calculated starting from the corresponding previous intermediate state.

Given the present 4-state model, six transition probabilities from a state to the other were possible (Fig. 2). The probability to transition from pre- to overt PMF, AML, and death accounted for 15.2, 4.7, and 17.3% of the 382 initial cases (Fig. 2, panels 1, 2, and 3, respectively). In panels 4–6, the probability of further transition of patients who had occupied an intermediate state is presented. The evolution into AML from a state of overt PMF was the highest in the first 5 years (around 7%), but then leveled off at <1% (panel 4). Conversely, the probability of death after an overt PMF or leukemic progression (panels 5 and 6, respectively) increased much more rapidly over time, especially when compared to the probability to die from a pre-PMF state without disease progression (panel 3).

**Fig. 3:**
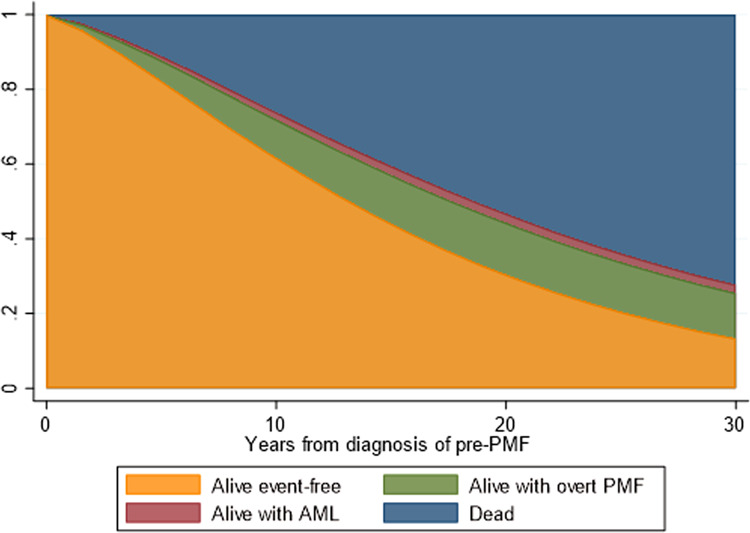
Stacked plot of state occupation probabilities of being alive, free from evolution, being alive with overt PMF, alive with AML, or having died as a function of time since diagnosis of pre-PMF (years). AML acute myeloid leukemia, PMF primary myelofibrosis. The figure shows the state occupation probabilities, which are the probability of being in a state at a certain time. The stacked presentation allows to compare the four different probabilities simultaneously. At time 0 (pre-PMF diagnosis), the probability of being alive and free from evolution is 100%. During the course of the disease, this probability gradually decreases in favor of other state occupations. In particular, the probability of intermediate-state occupation is very low for AML (1–2%) and higher for overt PMF (15–20%). The probability of being in the final state (death) increases much more rapidly from 10 years after pre-PMF diagnosis, to reach around 60% at 20 years.

The time duration of state occupation, reported in the stacked plot of Fig. 3, gradually decreased over time for patients with pre-PMF with an approximately linear trend; 70% and 30% of these patients remained event free after 10–20 years, respectively; in the same period, the probability of death increased from 30% to 60% at 10 and 20 years, respectively. State occupation probabilities for intermediate states (overt PMF and AML, considered together) gradually increased over time and remained relatively stable (20–22%) after 5–10 years.

### Prognostic factors predicting direct transitions to overt PMF, AML, and death

We searched for prognostic factors for transitions 1, 2, and 3 (the direct pathway from pre-PMF to evolution to overt PMF, AML, and death, respectively). Estimates of covariate effects in each transition-specific model are reported in Table 2. Clinical covariates, including sex, age, blood values, fibrosis grade, spleen size, LDH, and abnormal cytogenetics, also corrected for therapy, were considered in the first model, whereas the prognostic value of mutational findings (driver as well as other mutations) was separately evaluated as the latter were available in only a fraction of the entire population (*n* = 132 out of 382 patients).

**Table 2:** Covariate effect in each transition-specific model.

Covariate	Transition 1: evolution in overt PMF		Transition 2: evolution in AML		Transition 3: death	
	HR (95% CI)	*P*	HR (95% CI)	*P*	HR (95% CI)	*P*
***Clinical***^a^						
Male sex	0.91 (0.44–1.87)	0.790	0.34 (0.08–1.46)	0.147	1.66 (0.64–4.26)	0.296
Age >65 years	0.56 (0.25–1.30)	0.176	**10.3 (2.33–45.6)**	**0.002**	**6.53 (2.38–17.9)**	**<0.0001**
Anemia^b^	**2.18 (1.01–4.72)**	**0.047**	1.34 (0.31–5.92)	0.695	2.30 (0.88–5.37)	0.153
WBC > 15 × 10^9^/L	0.91 (0.29–2.84)	0.870	**4.80 (1.22–18.9)**	**0.025**	**3.49 (1.35–9.01)**	**0.010**
Plt > 1000 × 10^9^/L	1.96 (0.64–5.97)	0.236	0.47 (0.07–3.16)	0.438	1.21 (0.30–4.84)	0.786
Fibrosis grade 1	**3.20 (1.08–9.41)**	**0.035**	0.34 (0.09–1.29)	0.112	2.96 (0.82–10.7)	0.100
Spleen size > 5 cm	0.59 (0.17–2.05)	0.404	2.59 (0.66–10.2)	0.175	0.79 (0.25–2.53)	0.694
LDH > 1.5	1.58 (0.76–3.30)	0.221	**8.13 (1.46–45.2)**	**0.017**	0.84 (0.36–1.98)	0.687
Abnormal cytogenetics	1.60 (0.64–4.00)	0.310	**6.92 (1.61–29.8)**	**0.009**	1.84 (0.71–4.76)	0.209
***Mutational***^***c***^						
*Driver*						
*JAK2*V617F	0.87 (0.04–17.4)	0.925	0.12 (0.02–6.00)	0.286	0.82 (0.07–10.2)	0.875
*MPL*W515L/K	0.93 (0.06–14.4)	0.955	0.52 (0.01–24.7)	0.740	1.91 (0.21–17.8)	0.570
*CALR*	0.91 (0.06–14.4)	0.955	0.13 (0.02–7.27)	0.319	0.36 (0.04–3.68)	0.390
Triple negatives	2.70 (0.10–74.3)	0.557	0.13 (0.00–10.6)	0.368	3.31 (0.23–46.6)	0.376
*Nondriver*						
HMR^d^	**3.15 (1.08–9.21)**	**0.036**	–	–	**4.62 (2.09–10.2)**	**<0.0001**
HMR^e^ 2	–	–	**6.62 (1.11–39.3)**	**0.038**	–	–

All transitions assume a Weibull baseline function.

*HR* hazard ratio, *CI* confidence interval, *Hb* hemoglobin, *Hct* hematocrit, *Plt* platelets, *WBC* white blood cell, *LDH* lactate dehydrogenase, *HMR* high molecular risk

^a^Multivariate models also corrected for treatment effect (chemotherapy yes/no; antiplatelets yes/no; anticoagulants yes/no).

^b^Anemia was defined for values of Hb < 12 g/dL for females and Hb < 13 g/dL for males.

^c^Multivariate models best fitted with HMR or HMR 2 alternatively.

^d^HMR, high molecular risk, points to the presence of at least one mutation in any one of ASXL1, EZH2, SRSF2, and IDH1/2.

^e^HMR 2 means the presence of 2 or more mutated genes among the above. Two or more mutations in the same gene are counted as one.

Significant *P*-values (*P* < 0.05) are indicated in bold.

Independent significant clinical variables associated with the transition from pre- to overt PMF were grade 1 BM reticulin fibrosis (HR = 3.20, *P* = 0.035) and anemia (HR = 2.18, *P* = 0.047). Variables associated with the transition into AML were age >65 years (HR = 10.3, *P* = 0.002), leukocytosis defined as white blood cell (WBC) count > 15 × 10^9^/L (HR = 4.80, *P* = 0.025), LDH ratio > 1.5 times the upper normal value (HR = 8.13, *P* = 0.017), and cytogenetic abnormalities (HR = 6.92, *p* = 0.009). Finally, transition to death was associated with age >65 years (HR = 6.53, *P* < 0.0001) and leukocytosis of >15 × 10^9^/L (HR = 3.49, *P* = 0.010).

Concerning models including mutational status, the presence of one or more HMR mutations was the only factor significantly associated with an increased risk of transition from pre- to overt PMF (HR = 3.15, *P* = 0.036), AML (HR = 6.62, *P* = 0.036), and death (HR = 4.62, *P* < 0.0001).

**Fig. 4:**
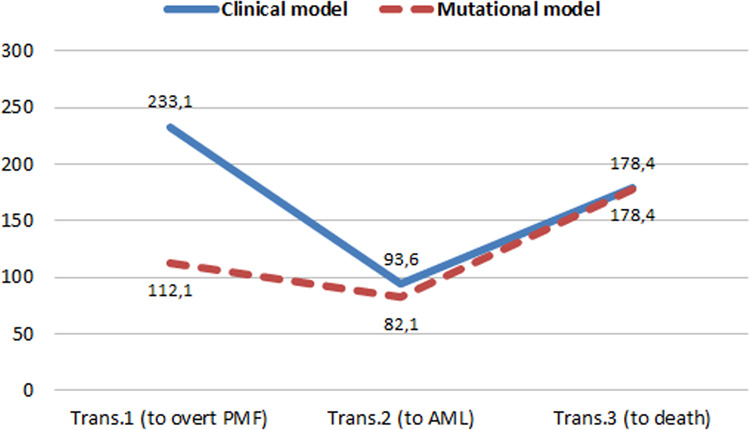
Akaike’s information criterion (AIC) of clinical and mutational models for direct transitions. Trans transition, AML acute myeloid leukemia, PMF primary myelofibrosis. The figure compares the prognostic performance of clinical and mutational models for the prediction of the 3 direct transitions (overt PMF, AML, or death) by their AIC values. The lowest AIC corresponds to better performance.

The Akaike’s information criterion (AIC) was then calculated for both clinical and mutational models in order to compare their prognostic performance across the 3 direct transitions (Fig. 4). Superimposable values of AIC were obtained for transitions 2 and 3 (to AML and death, respectively) (Fig. 4). On the contrary, a better prognostic value of transformation from the pre- to overt PMF status was found for the model including HMR mutations, where AIC was lower (AIC = 112) for the mutational model in comparison to the model considering only clinical covariates (AIC = 233).

## Discussion

This study outlines both estimates of the probability of transition from one state to another and risk factors affecting the transition of pre- to overt PMF, AML, and death.

### Transformation to overt PMF

This transformation occurred in 15.2% of subjects overall, over a median follow-up of nearly 7 years. This evolution pattern is in general agreement with previous observations, provided the different methods of selection from filed material were taken into account^4,6–8,15^. A bias might occur if reclassification is focused either on patients presenting with essential thrombocythemia (ET) or PMF. Consequently, cohorts with a more “ET-like phenotype” (higher platelet counts > 450 × 10^9^/L, lower LDH levels, splenomegaly < 5 cm, and fewer peripheral blasts) versus groups with a more “PMF-like phenotype” (lower platelet counts, higher LDH levels, more pronounced splenomegaly, and higher peripheral blood%) may be recognized^16^. In two studies investigating explicitly patients presenting with the original diagnosis of ET that were later reclassified by applying the WHO criteria^1,2^ as pre-PMF with thrombocythemia, comparable risk rates were revealed. The first study^4^ reported a cumulative incidence of overt PMF at 10 years of 12.3% and the other^8^ of 9.7%. As could be expected by the corresponding clinical data, higher-frequency rates of pre-PMF than in ET-like phenotypes were found when selecting from a cohort with the former diagnosis of PMF^6^. Moreover, our results from this multistate analysis should be considered more reliable since they take into account the effects of the competing absorbing states of death and direct AML evolution. Two independent clinical prognostic risk factors were identified to predict evolution into overt PMF, i.e., anemia (hemoglobin less than 12 g/dL for females and 13 g/dL for males) and grade 1 BM reticulin fibrosis^17^. In addition, whereas driver mutations showed no association with transition to overt PMF, the presence of at least one of HMR mutations (*ASXL1, EZH2, SRSF2*, and *IDH1/2*) was independently associated with this event.

### Transformation to AML

In prefibrotic PMF, several groups have reported variable frequencies concerning 10-year cumulative incidences of AML, ranging from 2.3^8^, 5.8^4^, and 12%^6^. In this context, it is important to show that evolution from pre-PMF to AML may follow two different pathways, as shown in Fig. 1: one direct (64% of cases) and one preceded by an intermediary state of overt PMF (36% of cases). In the direct pathway, the probability to appreciate the occurrence of evolution into AML is fairly stable up to 10 years (Fig. 2), and increases afterward. Conversely, in the indirect pathway, i.e., once myelofibrotic evolution has occurred, evolution in AML is more likely to happen in the first 10 years and decreases afterward. This is not surprising, as it is expected that the probability of AML is higher in overt PMF; yet, roughly two-thirds of transformations do not pass through overt PMF, a fact that should not be underestimated in clinical practice.

Clinical and mutational risk factors were found to affect direct transformation from pre-PMF to AML. Patients aged 65 or more, leukocytosis (WBC > 15 × 10^9^/L), LDH ratio > 1.5, and cytogenetic abnormalities were independently associated with AML evolution. The presence of this pattern calls for careful initial evaluation of this subcategory of patients since diagnosis, as well as for continuous monitoring. While the prognostic value of driver mutations was limited, the risk of AML transition was higher in patients with at least one HMR mutation (i.e., *ASXL1, EZH2, SRSF2*, and *IDH1/2*) and further increased with two or more such mutations.

### Death

Over a median follow-up of approximately 7 years, 105 deaths were registered, corresponding to 27% of the study population (*n* = 382). While conventional statistical analysis produces estimates of survival by considering directly the final event, such as death, the multistate model provides a more reliable estimate of survival since it involves in the calculation the intermediate states (PMF and AML) as well. For example, we would not know, by standard Kaplan–Meier survival analysis alone, whether these deceased patients were blastic or fibrotic at the time of death. This model allowed us to separate the influence of AML and overt PMF from mortality related to stable pre-PMF alone. Our analysis showed that, while mortality is higher after disease progression, as expected, in absolute terms, the greatest part of deaths happens in progression-free patients (64%). This mortality appears to be excessive for patients of that age, and is most likely accounted for by some independent prognostic factors, the most relevant ones being leukocytosis and the presence of at least one HMR mutation. We stress that leukocytosis, in our model, is a risk factor for death independently from its known effect on disease progression.

### Limitations

We acknowledge that our work has some limitations. In particular, in the analysis of prognostic factors, mutational and clinical risk factors were assessed separately and not together as they should have been. This was due to the limited number of patients in whom information on HMR mutations was available (only a third of the study population was informative in that regard). This prevents the possibility of developing a full-fledged prognostic score to differentiate the risks in subgroups of patients. However, in addition to demonstrating also with our multistate model that driver mutations do not influence transitions from pre-PMF to other states, our results highlight an adverse prognostic role of HMR mutations, confirming previous observations. We also specified that comparisons of AIC between the mutational and clinical model were in favor of the mutational model only for transformation to overt PMF, whereas for the other transitions, AIC was basically superimposable.

In conclusion, besides providing epidemiological estimates of the probability of transition from one disease phase to another, in patients with pre-PMF, this study traces the clinical course of the disease, allowing prognostication of the most likely path in each patient, and facilitates a more appropriate therapy decision-making.
